# Central venous catheter infection with Bacillus pumilus in an immunocompetent child: a case report

**DOI:** 10.1186/1476-0711-6-12

**Published:** 2007-10-29

**Authors:** HN Bentur, AM Dalzell, FAI Riordan

**Affiliations:** 1 Department of Paediatric Gastroenterology, Royal Liverpool Children's Hospital (Alder Hey), Liverpool, UK; 2 Department of Paediatric Infectious Diseases, Royal Liverpool Children's Hospital (Alder Hey), Liverpool, UK

## Abstract

**Background:**

Bacillus organisms are common laboratory contaminants. The majority of Bacillus bacteraemias are transient and not clinically significant. Clinically significant infection due to Bacillus species is rare and mostly due to Bacillus cereus infections in immuno-compromised hosts.

**Case presentation:**

We report a case of central venous catheter infection with Bacillus pumilus in an immunocompetent child with tufting enteropathy on long-term parenteral nutrition (PN). There were three episodes of central venous catheter infection with Bacillus pumilus in three months. Despite adequate and appropriate use of intravenous antibiotics, the infection failed to clear resulting in the need for removal of the catheter for complete cure.

**Conclusion:**

Bacillus species can cause clinically significant central venous catheter infection, even in an immunocompetent host. Despite adequate antibiotic treatment, the central venous catheter may need removal for complete cure.

## Background

Central venous catheter infections are a major cause of morbidity [1-3]. In most cases, the offending organisms are coagulase-negative staphylococci, Staphylococcus aureus, gram-negative bacilli, and Candida species.

Bacillus species are common contaminants of blood cultures but clinically significant infection is rare [4,5]. Significant central venous catheter infections caused by Bacillus species are mainly due to B cereus and have mostly been reported in immunocompromised hosts [6,7]. We report a case of central venous catheter infection with Bacillus pumilus, in an immunocompetent child with tufting enteropathy on long-term parenteral nutrition.

## Case report

An 8 year old girl with tufting enteropathy on long-term parenteral nutrition presented on 3 occasions with central venous catheter infection due to Bacillus species. On each occasion, she had fever after flushing of the central venous catheter. She had initially presented in the first few months of life with chronic watery diarrhoea and impaired growth, and was found to have tufting enteropathy (intestinal epithelial dysplasia) [8]. This is a rare congenital enteropathy, which requires indefinite dependence on parenteral nutrition from early infancy. The child is on regular parenteral nutrition and has had no previous history of significant infections, except for central venous catheter infections with coagulase negative staphylococci. Immunoglobulins, neutrophil and lymphocyte counts were within the normal range. There was no history of significant trauma, injuries or skin infections prior to this episode, except a small cut on her finger which healed very well and was generally well in herself. She lives with her parents and is well cared for. There is no history of contact with plant growth products or animal probiotics at any time.

### First episode

The child presented with fever and rigors to her local hospital. Bacillus species was isolated from blood taken from the central venous catheter, which was reported sensitive to flucloxacillin. She was treated with 4 weeks of intravenous flucloxacillin because bacteraemia had persisted despite 14 days of treatment.

### Second episode

The child was transferred to our hospital with recurrence of fever and rigors, 10 days after stopping the antibiotics. Empirical treatment was started with intravenous cefotaxime and flucloxacillin. Bacillus species was isolated from central venous catheter cultures both before and whilst on cefotaxime and flucloxacillin. This was later identified as Bacillus pumilus at the National Reference Laboratory (Health Protection Agency, Centre For Infection, London). The methods used to identify the organism were gram stain to determine whether spores are produced, short biochemical profile based on ammonia salt sugars, Lecithinase and mannitol (B. pumilus is lecithinase negative and mannitol positive) and DNA sequencing. *B pumilus *was reported to be sensitive to vancomycin and erythromycin. There were concerns that the patient had previously reacted to systemic vancomycin, so antibiotics were changed to intravenous clindamycin with vancomycin line locks given for 2 weeks. Blood cultures, taken both during and after this treatment, were negative. Echocardiography showed no evidence of vegetations at the tip of the catheter or in the heart.

### Third episode

Ten days after stopping the intravenous antibiotics the child presented for the third time with fever and rigors. A Bacillus species was again grown from blood taken from the central venous catheter. The central venous catheter was removed after 5 days treatment with intravenous vancomycin and a new central venous catheter was inserted. Subsequent blood cultures were negative and there has been no recurrence of further fever or infections over a 9-month period, suggesting the infection has been eradicated.

## Discussion

Central Venous Catheter infection is an important cause of morbidity in children dependent upon central venous catheters. In about two-thirds of cases, the offending organisms are coagulase-negative staphylococci, Staphylococcus aureus and gram negative bacilli. Candida species are responsible for one-third of these infections and carry a worse prognosis [1-3]. Currently there are no published data on the frequency with which Bacillus species are recovered from central venous catheters, but clinically significant infection due to Bacillus species are rare.

Most reports of infections caused by Bacillus species have occurred in intravenous drug users, those with prosthetic valvular devices and in immuno-compromised hosts [9]. Bacillus species grown from blood are frequently assumed to be contaminants. Bacillus species are isolated from 0.1–0.9% of blood cultures, but only 5–10% of these are clinically significant infections [4,5]. Since Bacillus isolates are frequently dismissed as contaminants most laboratories do not identify these isolates further. Infection due to Bacillus pumilus has thus been rarely reported.

The genus Bacillus consists of a heterogenic group of gram-positive, endospore-forming, rod-shaped, facultative anaerobic bacteria [10]. Bacillus species other than Bacillus anthracis produce spores that are widespread in the environment, and isolation from a specimen may represent contamination. Of the non-anthrax Bacillus species, B. cereus, B. licheniformis, and B. pumilus may be more pathogenic in immunosuppressed hosts than other common Bacillus species, (B. subtilis or B. megaterium). However, Bacillus pumilus has rarely been reported as a human pathogen.

Risk factors for Bacillus bacteraemia include intravenous drug use [9] hemodialysis [11] and leukemia [12]. Intravascular catheters, pacemaker wires, skin or wound infections have all been reported as potential portals of entry of infection for bacteraemia with Bacillus [6,7]. Significant bacteraemia with Bacillus species has been described in immunosuppressed or cancer patients [[11-13] and [14]]. To our knowledge, there is one case report of Bacillus cereus infection of a central line in an immunocompetent patient (a child with haemophilia) [15]. It is unclear why our patient should have infection with Bacillus pumilus, although there may be some subtle immune dysfunction in tufting enteropathy in addition to malabsorption [8].

Bacillus pumilus has toxic properties; it has cytopathic effects in Vero cells, haemolytic activity, lecithinase production, and proteolytic action on casein [16,17]. The organism produces a toxin that has been detected in guinea pigs with experimentally induced enterocolitis associated with clindamycin [13]. Bacillus pumilus is also known to be used in some plant growth products [18] and some of the commercially used animal probiotics [19].

Antibiotics, which appear useful in the treatment of non cereus Bacillus, are clindamycin, imipenem, ciprofloxacin and vancomycin, to which the vast majority of strains are susceptible [20,21]. Some suggest that initial antibiotic treatment should be vancomycin until susceptibilities are known. We did not use this initially in our case because of concerns about a possible previous reaction to vancomycin. However despite an adequate course of appropriate antibiotics the infection recurred as in previous reports of Bacillus central venous catheter infection [3]. Guidelines are not available for antibiotic testing of Bacillus species by routine disc susceptibility testing [22]. Tests to determine minimum inhibitory concentrations are therefore needed.

Central venous catheter infection with some organisms (e.g. coagulase negative staphylococci), can be successfully eradicated by antibiotics alone without resorting to catheter removal [3]. However, central venous catheter infection with Bacillus species usually requires catheter removal for complete cure, as in our case [1-3].

Our experience suggests that Bacillus species can cause clinically significant central venous catheter infections, even in an immunocompetent child. Bacillus species isolated from blood taken from central venous catheters should not be dismissed as contaminants; instead more blood cultures should be taken. Treatment should be instituted if these repeat cultures are positive or if the patient's deteriorates clinically. However, despite antibiotic treatment the central venous catheter may need removal for complete cure.

## Competing interests

The author(s) declare that they have no competing interests.

## Authors' contributions

HNB provided clinical care, literature search, initial drafting and editing of manuscript.

AMD provided clinical care, critical appraisal and editing of manuscript.

FAI R oversaw antimicrobial susceptibility testing and the antimicrobial regimen for this patient, helped conceive this paper, critical appraisal and editing of manuscript

All authors approved of final manuscript submission.
